# Feasibility of biohydrogen production from industrial wastes using defined microbial co-culture

**DOI:** 10.1186/s40659-015-0015-x

**Published:** 2015-05-06

**Authors:** Peng Chen, Yuxia Wang, Lei Yan, Yiqing Wang, Suyue Li, Xiaojuan Yan, Ningbo Wang, Ning Liang, Hongyu Li

**Affiliations:** School of Pharmacy, Lanzhou University, Donggang West Road No. 199, Lanzhou, 730020 People’s Republic of China; Gansu Institute of Business and Technology, Yannan Road No. 449, Lanzhou, 730010 People’s Republic of China; Key Laboratory of Fermentation Resources and Application of Institutes of Higher Learning in Sichuan, School of Life Science and Food Engineering, Institute for Bioengeering, Yibin University, Jiusheng Road No. 8, Yibin, 644000 People’s Republic of China; College of Life Science and Technology, Heilongjiang Bayi Agricultural University, Daqing, 163319 People’s Republic of China; The Reproductive Medicine Research Center of the First Hospital of Lanzhou University, Donggang West Road No. 1, Lanzhou, 730020 People’s Republic of China

**Keywords:** Renewable Energy, Biohydrogen, Microbial consortium, Hydrogen

## Abstract

**Background:**

The development of clean or novel alternative energy has become a global trend that will shape the future of energy. In the present study, 3 microbial strains with different oxygen requirements, including *Clostridium acetobutylicum* ATCC 824, *Enterobacter cloacae* ATCC 13047 and *Kluyveromyces marxianus* 15D, were used to construct a hydrogen production system that was composed of a mixed aerobic-facultative anaerobic-anaerobic consortium. The effects of metal ions, organic acids and carbohydrate substrates on this system were analyzed and compared using electrochemical and kinetic assays. It was then tested using small-scale experiments to evaluate its ability to convert starch in 5 L of organic wastewater into hydrogen. For the one-step biohydrogen production experiment, H1 medium (nutrient broth and potato dextrose broth) was mixed directly with GAM broth to generate H2 medium (H1 medium and GAM broth). Finally, *Clostridium acetobutylicum* ATCC 824, *Enterobacter cloacae* ATCC 13047 and *Kluyveromyces marxianus* 15D of three species microbial co-culture to produce hydrogen under anaerobic conditions. For the two-step biohydrogen production experiment, the H1 medium, after cultured the microbial strains *Enterobacter cloacae* ATCC 13047 and *Kluyveromyces marxianus* 15D, was centrifuged to remove the microbial cells and then mixed with GAM broth (H2 medium). Afterward, the bacterial strain *Clostridium acetobutylicum* ATCC 824 was inoculated into the H2 medium to produce hydrogen by anaerobic fermentation.

**Results:**

The experimental results demonstrated that the optimum conditions for the small-scale fermentative hydrogen production system were at pH 7.0, 35°C, a mixed medium, including H1 medium and H2 medium with 0.50 mol/L ferrous chloride, 0.50 mol/L magnesium sulfate, 0.50 mol/L potassium chloride, 1% w/v citric acid, 5% w/v fructose and 5% w/v glucose. The overall hydrogen production efficiency in the shake flask fermentation group was 33.7 mL/h^-1^.L^-1^, and those the two-step and the one-step processes of the small-scale fermentative hydrogen production system were 41.2 mL/h^-1^.L^-1^ and 35.1 mL/h^-1^.L^-1^, respectively.

**Conclusion:**

Therefore, the results indicate that the hydrogen production efficiency of the two-step process is higher than that of the one-step process.

## Background

The research and development of hydrogen energy has attracted worldwide attention and become an important strategy for the production of clean energy [1,2]. Among the various technologies that have been developed for hydrogen production, the utilization of hydrogen-producing microbes is one of the most effective. Currently, microbial fermentative hydrogen production is achieved mainly through 2 routes: one utilizes pure microbial strains and the other employs a mixed microbial consortium [3-6]. Researchers have performed a large number of studies on fermentative hydrogen production using pure microbial strains and its disadvantages, including short test cycles, low hydrogen yields and poor applicability in the presence of complex substrates. To take better advantage of low-cost organic substrates for hydrogen production, recycle wastes and improve the environment, in recent years, many researchers have focused on hydrogen production by microbial fermentation using a mixed microbial consortium, in which various microbial strains exert synergistic effects [7,8]. In a favorable living environment that fully supports metabolic activities, the hydrogen production capacity and hydrogen yield from the mixed microbial consortium were enhanced compared with those of the pure microbial strains. Recent studies have confirmed that the hydrogen production efficiency of mixed microbial consortia are significantly higher than that of individual pure strains [9,10]. Therefore, the production of hydrogen by fermentation using low-cost agricultural waste by a mixed microbial consortium is a novel development for the hydrogen energy industry [11].

Our previous work was to observe the hydrogen production yield of 16 different pure strains and mixed culture, which contains hydrogen-producing strains and non-hydrogen-producing strains. The experimental results compared the hydrogen production yield of these pure strains. Based on these results and on the comparative hydrogen-production efficiencies of the microbial strains, *Enterobacter cloacae* (*E. cloacae*) ATCC 13047, *Kluyveromyces marxianus* (*K. marxianus*) 15D and *Clostridium acetobutylicum* (*C. acetobutylicum*) ATCC 824 were identified as suitable for use in hydrogen-producing mixed microfloras. In the present study, by the application of combinatorially optimized culture media, 3 microbial strains with different oxygen requirements, including *C. acetobutylicum* ATCC 824, *E. cloacae* ATCC 13047 and *K. marxianus* 15D, were combined to construct a hydrogen production system that was composed of a mixed aerobic-facultative anaerobic-anaerobic consortium.

The metal ions, organic acids and carbohydrate substrates are the most common material in industrial wastes environment. The ability of microorganism to produce hydrogen in the presence of industrial wastes sources (metal ions, organic acids and carbohydrate substrates) is worthy of study. The fundamental knowledge derived from this study should provide a valuable platform for further investigation into the behavior of microorganism involved in hydrogen production system and has potential biotechnological applications in waste resources reused. Therefore, the effects of metal ions, organic acids and carbohydrate substrates on the system were analyzed by assaying the electrochemical and kinetic parameters, along with the optimization and quality control of hydrogen production process. Based on the identification of the factors affecting the hydrogen production process, a small-scale biohydrogen production system (5 L) using starchy organic wastewater was constructed with the intent of providing preliminary data for further pilot-scale biohydrogen production.

## Results and discussion

### Effects of metal ions on hydrogen production system composed of mixed microbial consortium

To determine the effects of various metal ions on the H1 medium- and H2 medium-based hydrogen production systems, a two-step system that was composed of the 3 microbial strains was studied. The experimental results are shown in Table 1. In this experiment, the controls correspond to cultures with no supplemented metal ions. Table 1 demonstrates that Fe^2+^ had stimulatory effects on the hydrogen production system that was composed of the mixed microbial strains *E. cloacae* ATCC 13047 and *K. marxianus* 15D, while Fe^3+^ activated the system that was composed of the bacterial strain *C. acetobutylicum* ATCC 824. Previous studies have shown that at the cellular level, certain metal ions affect the activity and quantity of hydrogen-producing bacteria. It has been shown that iron deficiency not only affects the growth and metabolism of the ethanol-based hydrogen-producing fermentative bacterium B49 but also its ability to produce hydrogen. The addition of Fe^2+^ to a culture of hydrogen-producing fermentative bacteria has been shown to stimulate the specific activities of hydrogenase and NADH-Fe reductase, enhancing its hydrogen production activities and improving its hydrogen production capacities [12].

**Table 1:** **The effects of different metal ions on the hydrogen production systems**

**Metal ions (0.50 mol/L)**	**H1 medium (mL/h** ^**-1**^ **.L** ^**-1**^ **)**	**H2 medium (mL/h** ^**-1**^ **.L** ^**-1**^ **)**	**Overall hydrogen production (%)**
Controls	17.80 ± 1.51	15.90 ± 0.66	100.0
BaCl_2_ · 2H_2_O	3.74 ± 1.04	2.07 ± 0.36	17.21
CaCl_2_	6.23 ± 0.25	4.29 ± 1.03	31.16
CoCl_2_ · 6H_2_O	2.14 ± 0.99	1.113 ± 1.04	9.50
FeCl_2_ · 4H_2_O	21.54 ± 1.02	13.67 ± 0.76	104.45
FeCl_3_ · 4H_2_O	14.06 ± 1.09	18.44 ± 0.58	96.44
CuSO_4_ · 5H_2_O	14.77 ± 1.17	10.65 ± 1.97	75.37
KCl	18.69 ± 0.23	15.90 ± 1.54	102.67
MnSO_4_ · H_2_O	13.53 ± 0.76	10.02 ± 0.15	69.73
MgSO_4_ · 7H_2_O	21.18 ± 0.16	20.19 ± 0.83	122.85
NH_4_Cl	12.64 ± 1.88	10.81 ± 1.96	69.73
ZnSO_4_ · 7H_2_O	16.20 ± 1.09	12.08 ± 1.03	83.98

Static fermentation experiments using 20 hydrogen-producing bacterial strains have shown that the addition of ferrum to culture media causes a shift from butyric acid to ethanol fermentation. In the 2 main pathways of fermentative hydrogen production from organic materials, ferrum is one of the essential components that participate in and promote various enzymatic reactions. Under similar culture conditions, both Fe^3+^ and Fe^2+^ stimulate the conversion of bacterial metabolism to ethanol-type fermentation, and Fe^3+^ exhibits stronger effects than Fe^2+^. Furthermore, Fe^2+^ enhances the fermentative hydrogen production capacities of bacteria [13].

In addition, Mg^2+^ and K^+^ ions also exhibit stimulatory effects on hydrogen production systems. Mg^2+^ is an important influential factor. Of the 10 types of cytoplasmic enzymes that are required for glycolysis, a vast majority are activated by Mg^2+^, and its deficiency affects the growth and anabolism of hydrogen-producing bacteria, thereby affecting the ability of the bacteria to produce hydrogen. The addition of Mg^2+^ to culture media has been shown to promote the growth of ethanol-based hydrogen-producing fermentative bacteria and thus enhance their hydrogen production capacities [14]. In contrast, other metal ions, such as Ba^2+^, Ca^2+^, Co^2+^, Cu^2+^, Mn^2+^ and Zn^2+^, appear to exert inhibitory effects on hydrogen production systems by hindering microbial growth and hydrogenase activities.

### Effects of organic acids on hydrogen production systems composed of mixed microbial consortium

To determine the effects of various organic acids on H1 medium- and H2 medium-based hydrogen production systems, a two-step system that was composed of the 3 microbial strains was studied. Table 2 shows that citric acid significantly enhances hydrogen production efficiencies, whereas the remaining organic acids that were tested displayed inhibitory effects.

**Table 2:** **The effects of various organic acids on the hydrogen production efficiencies**

**Organic acids (1% w/v)**	**H1 medium (mL/h** ^**-1**^ **.L** ^**-1**^ **)**	**H2 medium (mL/h** ^**-1**^ **.L** ^**-1**^ **)**	**Overall hydrogen production (%)**
Controls	17.80 ± 1.51	15.90 ± 0.66	100.0
Acetic acid	6.20 ± 2.70	7.60 ± 1.60	40.95
Citric acid	18.0 ± 1.10	20.3 ± 1.02	113.65
Ethacetic acid	8.10 ± 3.12	8.0 ± 2.15	47.77
Lactic acid	19.0 ± 2.03	10.1 ± 1.02	86.35
Oxalic acid	12.0 ± 3.98	12 ± 1.25	71.22

### Effects of carbohydrate substrates on hydrogen production systems composed of mixed microbial consortium

To determine the effects of various carbohydrate substrates on H1 medium- and H2 medium-based hydrogen production systems, a two-step system that was composed of the 3 microbial strains was studied. As shown in Table 2, both fructose and glucose all exert significant stimulatory effects on the system, whereas the other carbohydrate substrates used in this study exhibited strong inhibition.

### Small-scale biohydrogen production experiments based on starchy organic wastewater

#### Construction of mixed microbial consortium for biohydrogen production and synergistic effects between microbial strains

**Figure 1:**
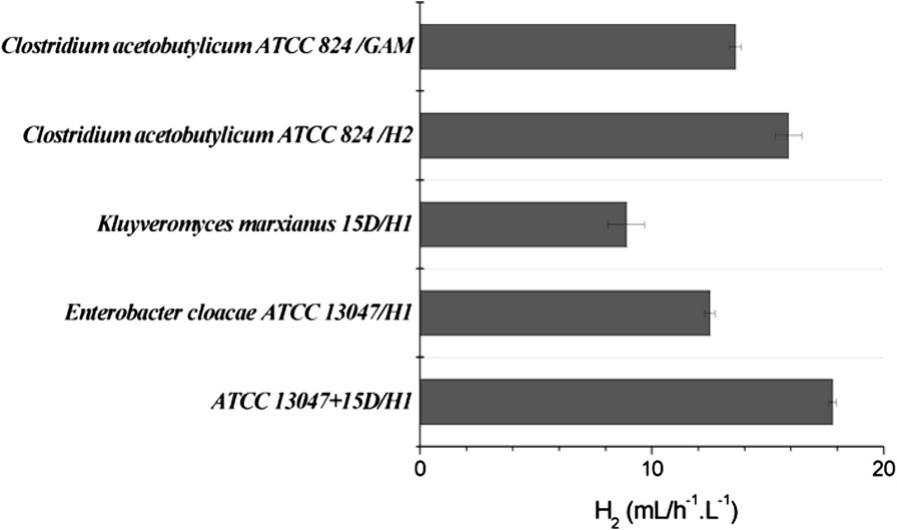
Analysis and comparison of the hydrogen production efficiencies of the mixed microbial consortia.

**Figure 2:**
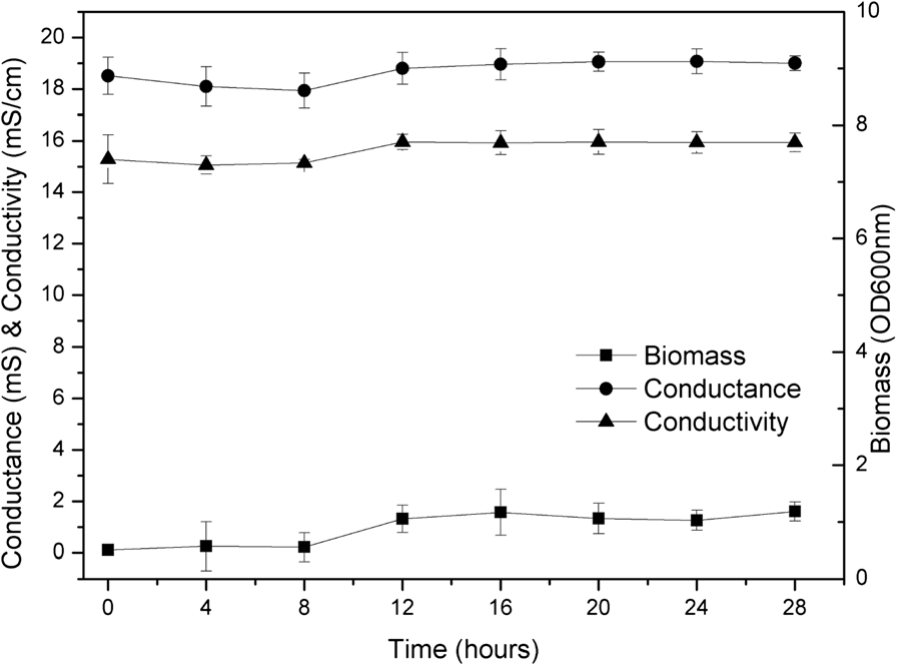
The time course of conductance and conductivity along with the optical density (OD) at 600 nm.

The biohydrogen-producing mixed microbial consortium was constructed using 3 hydrogen-producing microbial strains, including *C. acetobutylicum* ATCC 824, *E. cloacae* ATCC 13047 and *K. marxianus* 15D, and its electrochemical characteristics were analyzed. The hydrogen production efficiency of the mixed aerobic-facultative anaerobic consortium was found to be significantly higher than those of the hydrogen production systems that were composed of individual pure microbial strains (Figure 1). The hydrogen production efficiencies of *E. cloacae* ATCC 13047 and *K. marxianus* 15D were 12.5 mL/h^-1^.L^-1^ and 8.9 mL/h^-1^.L^-1^, respectively, whereas that of the mixed microbial consortium that was composed of the 2 microbial strains reached 17.8 mL/h^-1^.L^-1^. Therefore, the efficiency of the mixed microbial consortium was significantly enhanced compared to those of the pure microbial strains. The key determinant of whether a culture medium (H2 medium) for the aerobic-facultative anaerobic-anaerobic consortium could be successfully developed was whether the H1 medium that was harvested from the cultured microbial stains *E. cloacae* ATCC 13047 and *K. marxianus* 15D was able to support the growth of the bacterial strain *C. acetobutylicum* ATCC 824. Thus, *C. acetobutylicum* ATCC 824 was inoculated into the H2 liquid medium, and the electrochemical parameters of the culture were determined. The hydrogen production efficiency of *C. acetobutylicum* ATCC 824 in H2 medium was found to be significantly higher than that of the hydrogen production system that was composed of the pure culture of *C. acetobutylicum* ATCC 824. As shown in Figure 2, the efficiency of *C. acetobutylicum* ATCC 824 was 13.6 mL/h^-1^.L^-1^ in GAM broth, whereas it was 15.9 mL/h^-1^.L^-1^ in H2 medium. The increased efficiency that was observed in the H2 medium was probably due to the efficient degradation of starch in the H1 medium by the microbial strains *E. cloacae* ATCC 13047 and *K. marxianus* 15D. The H2 medium was constructed using the H1 medium as a base. Therefore, *C. acetobutylicum* ATCC 824 was able to better utilize the substrates, effectively converting glucose and other substances to hydrogen via the catalytic activity of hydrogenase. Thus, the efficiency of the two-step hydrogen production system that was composed of the mixed microbial consortium was significantly higher than that of the systems that were composed of the pure anaerobic strains [15,16].

#### Effects of 2 types of biohydrogen production processes on electrochemical properties of hydrogen production systems

The electrochemical parameters of the two-step and one-step hydrogen production systems were compared (Figure 2). In hydrogen-production systems composed of pure microbial strains, when all other conditions are constant, the electrical conductance of the biohydrogen-producing solution is determined by the number of ions, their electric charges, and their mobility. The electrical conductance of the biohydrogen-producing solution is the sum of the electrical conductances of the various ions in the solution. Therefore, in the biological hydrogen-production process, the electrical conductance and conductivity can be utilised to determine the concentrations of the components in a hydrogen-production system and to optimise hydrogen-production conditions.

The results showed the following: (1) in the one-step hydrogen production system, a gradual declining trend in pH was observed, whereas in the two-step system, an opposite trend was observed; (2) in the one-step hydrogen production system, the redox potential showed a gradual rising trend, increasing from 25 mV to 89 mV. In contrast, the redox potential of the two-step system decreased gradually within a small range; (3) the electrical conductance and conductivity of the one-step hydrogen production system were higher than those of the two-step system.

#### Effects of 2 types of biohydrogen production processes on hydrogen production efficiency

**Figure 3:**
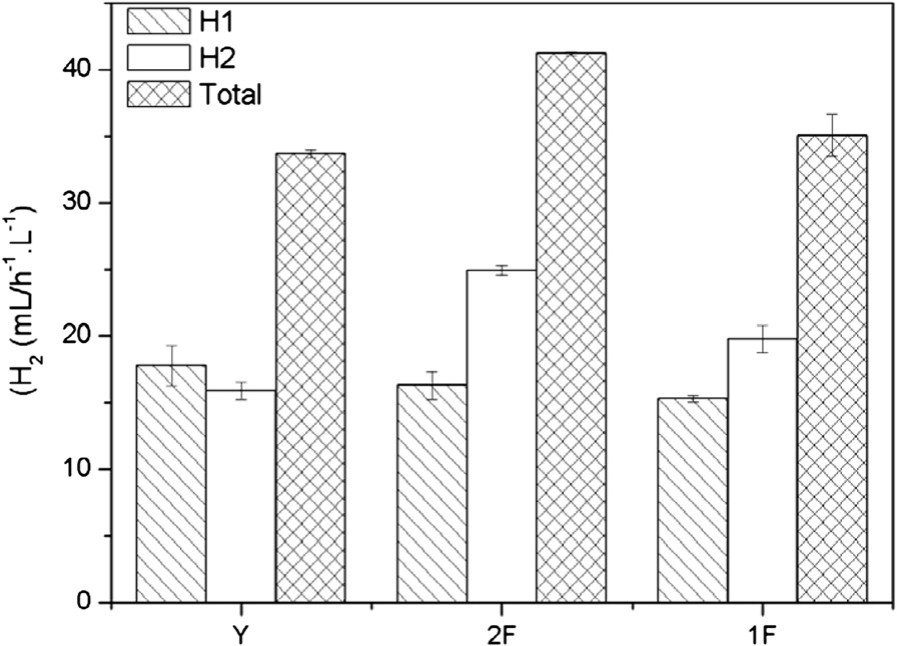
The effects of the 2 types of biohydrogen production processes on the hydrogen production efficiency.

The efficiencies of the 2 types of biohydrogen production processes were compared and analyzed. The results are shown in Figure 3. The shake flask fermentation group, which is abbreviated as Y in Figure 3, was used as the control. The two-step hydrogen production process is abbreviated as 2 F, and the one-step process is abbreviated as 1 F.

As shown in Figure 3, the overall hydrogen production efficiency in the shake flask fermentation group was 33.7 mL/h^-1^.L^-1^, and those the two-step and the one-step processes were 41.2 mL/h^-1^.L^-1^ and 35.1 mL/h^-1^.L^-1^, respectively, which were higher than that of the shake flask fermentation group. Therefore, the performance of the small-scale fermentative hydrogen production system was superior to the shake flask fermentation method. In addition, the overall hydrogen production efficiency of the two-step process was higher than that of the one-step process. The differences in the overall hydrogen production efficiencies were related to the removal of the centrifugation step.

### Microscopic analysis of mixed microbial consortium

**Figure 4:**
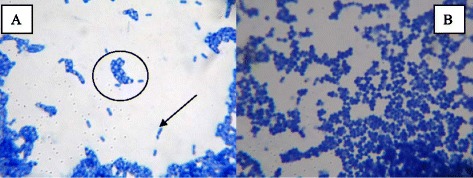
Microscopic morphologies of the microbial cells in mixed culture media **(A)** *C. acetobutylicum* ATCC 824 and *E. cloacae* ATCC 13047 in H1 medium; **(B)** *C. acetobutylicum* ATCC 824 in H2 medium.

The microscopic images of the mixed microbial consortium are shown in Figure 4. It was revealed that the microbial strains *E. cloacae* ATCC 13047 and *K. marxianus* 15D could be co-cultured in H1 liquid medium. *K. marxianus* 15D utilized starch for biomass production more efficiently than *E. cloacae* ATCC 13047. Therefore, compared to *E. cloacae* ATCC 13047 (Microbial shape is small rod, pointed out by the black arrow in Figure 4A), significantly larger numbers of *K. marxianus* 15D (Yeast shape is big sphere, denoted by the black circle in Figure 4A) were observed microscopically. Additionally, Figure 4B showed that the bacterial strain *C. acetobutylicum* ATCC 824 was capable of growing in H2 medium, and the amount of biomass that was produced by *C. acetobutylicum* ATCC 824 in the H2 medium (final OD_600nm_ = 2.3) was elevated compared to that in the GAM broth (final OD_600nm_ = 2.0). The results indicate that *C. acetobutylicum* ATCC 824 utilized the conditioned H1 liquid medium that was harvested from the culture of *E. cloacae* ATCC 13047 and *K. marxianus* 15D more efficiently, significantly improving the hydrogen production efficiency.

## Conclusion

The mixed microbial consortium was constructed by a two-step process using 3 microbial strains, including *C. acetobutylicum* ATCC 824, *E. cloacae* ATCC 13047 and *K. marxianus* 15D, and exhibited a high hydrogen production efficiency. Analyses of the effects of various metal ions, organic acids and carbohydrate substrates on the hydrogen production systems showed that the Fe^2+^/Fe^3+^ ions were crucial for the systems. Mg^2+^ and K^+^ also exerted stimulatory effects. Citric acid significantly enhanced hydrogen production efficiency. Additionally, fructose and glucose exhibited significant stimulatory effects. The hydrogen production efficiency of the two-step process is higher than that of the one-step process.

## Methods

### Microorganism

The microbial strains, used in the present study, were *C. acetobutylicum* ATCC 824, *E. cloacae* ATCC 13047 and *K. marxianus* 15D. All the three strains were preserved in our laboratory.

### Medium composition

#### The composition of the nutrient broth was as follows

10.0 g of peptone, 3.0 g of beef extract, 5.0 g of NaCl, 20.0 g of agar and 1.0 L of distilled water. The medium was adjusted to pH 7.0 with 5 mol/L sodium hydroxide (approximately 0.2 mL) and autoclaved at 1.05Kg/cm^2^ for 20 min.

The composition of the potato dextrose broth was as follows: 200.0 g of potato, 20.0 g of dextrose, 1.0 L of distilled water, pH 7.0. The potatoes were washed, peeled and sliced. The potato dextrose agar (PDA) medium was prepared from 200 g washed and sliced potatoes, boiled in 500 ml filtered and strained through gauze. 20.0 g Agar was melted in the solution, and 0.5 L water and 20.0 g glucose were added before the medium was aliquoted and autoclaved at 1.05Kg/cm^2^ for 20 min [17].

#### The aerobic-facultative anaerobic culture medium (abbreviation: H1 medium) was prepared as follows

The nutrient broth and potato dextrose agar were prepared separately as described above and mixed evenly. The medium was utilized for the routine culture of the microbial strains *E. cloacae* ATCC 13047 and *K. marxianus* 15D.

The composition of the modified Gifu anaerobic medium (GAM) broth was as follows: 15.0 g of proteose peptone, 10.0 g of pancreatic casein peptone, 5.0 g of yeast extract, 2.0 g of beef powder, 13.5 g of digestive serum powder, 1.2 g of bovine liver extract powder, 3.0 g of glucose, 2.5 g of potassium dihydrogen phosphate, 3.0 g of sodium chloride, 0.3 g of soluble starch, 0.3 g of L-cysteine and 0.15 g of sodium thioglycolate. The medium was prepared by adding 1.0 L of distilled water to 74.0 g of modified GAM broth and autoclaved at 1.05Kg/cm^2^ for 20 min.

#### The mixed culture medium (abbreviated: H2 medium) was prepared as follows

The microbial strains *E. cloacae* ATCC 13047 and *K. marxianus* 15D were inoculated into the H1 medium. After an incubation time of 30 h, the microbial cultures were centrifuged at 10, 000 r/min for 10 min, and the liquid medium were collected. The H2 medium was obtained by adding 74.0 g of modified GAM broth to 1.0 L of liquid medium, which was then autoclaved at 1.05Kg/cm^2^ for 20 min. The H2 medium was used to culture the bacterial strain *C. acetobutylicum* ATCC 824

#### Culture medium for the small-scale biohydrogen production system was prepared as follows

To produce biohydrogen, the microbial strains were cultured in H1 medium or H2 medium. The H1 medium contained additional components that were not present in the H1 medium, including 0.50 mol/L ferrous chloride, 0.50 mol/L magnesium sulfate, 0.50 mol/L potassium chloride, 1% w/v citric acid, 5% w/v fructose and 5% w/v glucose.

### Reagents

The reagents that were used in the present study included peptone (OXIDE), beef extract (BBI), agar (BBI), GAM broth (Qingdao Hi-tech Industrial Park Haibo Biotechnology Co., Ltd.), potatoes, corn flour (commercially available), starch (made in our laboratory from fresh potatoes), lactose, maltose, fructose, glucose and sucrose (all of the above carbohydrates were biochemical reagent grade). All other reagents were analytical grade. An anaerobic gas mixture that was composed of 5% (v/v) CO_2_, 10% (v/v) H_2_ and 85% (v/v) N_2_ was used as carrier gas. The N_2_ had a purity level of 99.999% (v/v). The content of impurities in N_2_ was as follows: H_2_, ppm (v/v) ≤ 1.0, O_2_, ppm (v/v) ≤ 3.0 and H_2_O, ppm (v/v) ≤ 5.0.

### Experimental procedures

#### Effects of various factors on hydrogen production systems

The H1 medium was prepared by adding various heavy metal ions, organic acids and carbohydrate substrates to the H1 medium in Tables 1, 2 and 3, respectively. The microbial strains *E. cloacae* ATCC 13047 and *K. marxianus* 15D were inoculated into the H1 medium, and samples were collected periodically during the incubation period to determine the electrochemical parameters. After an incubation time of 30 h, the microbial cultures were centrifuged, and the supernatants were mixed with modified GAM broth to generate the H2 medium. The bacterial strain *C. acetobutylicum* ATCC 824 was inoculated into the H2 medium and incubated anaerobically. Samples were collected periodically, and the electrochemical parameters were analyzed with the uninoculated H1 and H2 media as controls. The results presented were the average of three trials. The results are presented using mean value ± standard deviation in Tables 1, 2 and 3, respectively.

**Table 3:** **The effects of various carbohydrate substrates on the hydrogen production efficiencies**

**Carbohydrate substrates (5% w/v)**	**H1 medium (mL/h** ^**-1**^ **.L** ^**-1**^ **)**	**H2 medium (mL/h** ^**-1**^ **.L** ^**-1**^ **)**	**Overall hydrogen production (%)**
Controls	17.80 ± 1.51	15.90 ± 0.66	100.0
Fructose	23.3 ± 1.022	13.3 ± 1.94	108.61
Glucose	19 ± 1.22	16.1 ± 1.07	104.15
Lactose	16.4 ± 2.11	14.3 ± 1.04	91.10
Maltose	15.7 ± 2.01	15.5 ± 2.64	92.58
Sucrose	14.9 ± 2.56	16.7 ± 1.43	93.77
Starch	13.8 ± 2.44	17.3 ± 2.39	89.32

#### Small-scale biohydrogen production experiments using starchy organic wastewater

##### Construction of a two-step biohydrogen production system

In order to obtain an aerobic-facultative anaerobic-anaerobic hydrogen production system in a stepwise manner, the microbial strains *E. cloacae* ATCC 13047 and *K. marxianus* 15D were inoculated into the H1 medium. Samples were collected periodically during the incubation period to determine the electrochemical parameters. The H2 medium was constructed from the H1 medium that was harvested from the cultures of *E. cloacae* ATCC 13047 and *K. marxianus* 15D. The bacterial strain *C. acetobutylicum* ATCC 824 was then inoculated into the H2 medium and incubated anaerobically. Samples were collected periodically during the incubation period, and the electrochemical parameters were analyzed. Uninoculated H1 and H2 media served as controls, and the relevant parameters were determined.

For the two-step biohydrogen production experiment, the H1 medium, after cultured the microbial strains *E. cloacae* ATCC 13047 and *K. marxianus* 15D, was centrifuged to remove the microbial cells and then mixed with GAM broth (H2 medium). Afterward, the bacterial strain *C. acetobutylicum* ATCC 824 was inoculated into the H2 medium to produce hydrogen by anaerobic fermentation.

For the one-step biohydrogen production experiment, the centrifugation step in the two-step hydrogen production process was omitted to ensure the operability of this pilot-scale experiment. The H1 medium was mixed directly with GAM broth to generate H2 medium. The bacterial strain *C. acetobutylicum* ATCC 824 was inoculated into the H2 medium. Finally, three species microbial co-culture to produce hydrogen under anaerobic conditions.

##### Small-scale biohydrogen production experiments

The small-scale experiments were conducted in a 5 L fermentor. The experimental conditions were as follows: liquid volume of 3 L, fermentation temperature of 30°C, rotation speed of 200 r/min, ventilation rate of 0.5 L/min, inoculum volume of 10% and vessel pressure of 0.1 MPa. Samples were collected periodically. The yields of hydrogen were determined using the GC-9A gas chromatograph with a thermal conductivity detector (Shimadzu, Japan) with N_2_ as the carrier gas. The injection volume was 1 mL. Hydrogen concentrations were calculated using the external standard method.

The electrochemical parameters were measured using an FJA-3 electrochemical ion analyzer (Nanjing Zhuan-Di Instrument & Equipment Co., Ltd.). The fermentation broth was collected periodically, and the temperature, pH, Eh, electrical conductance and conductivity were detected. The measurements were repeated 5 times, and the averages were used for the data analysis. Using the uninoculated liquid medium as a reference, the absorbances of the microbial cultures at the 600 nm wavelength were measured using a UV-1800PC spectrophotometer (Beijing Purkinje General Instrument Co., Ltd.).

The microbial strains *E. cloacae* ATCC 13047 and *K. marxianus* 15D were inoculated into the H1 medium. Following the appropriate incubation period, the microbial cells were harvested by centrifugation, stained with methylene blue and imaged using the Canon Powershot A3300/S Digital Camera (Japan). The microscopes used in this study were the OPTON universal microscope (West Germany) and XSM-20 biological microscope (Ningbo Sunny Instruments Co., Ltd.).

### Analytical procedures

The hydrogen content was determined using a GC-9A gas chromatograph with a thermal conductivity detector (Shimadzu, Japan). The carrier gas was N_2_, and the injection volume was 1 mL. The hydrogen content was calculated using the external-standard method. Electrochemical parameters were measured using an FJA-3 electrochemical ion analyser (Nanjing Chuan-Di Instrument & Equipment Co., Ltd.). The fermentation broth was collected periodically, and the pH/Eh and electrical conductance/conductivity were determined. The measurements were repeated 5 times, and the average values were used for data analysis.
